# Interaction between CSL students’ motivation and anxiety under different L2 writing tasks: evidence from Vietnamese university students

**DOI:** 10.3389/fpsyg.2023.1230498

**Published:** 2023-11-23

**Authors:** Cong Wang, Sida Zhu, Haijing Zhang

**Affiliations:** ^1^Department of Chinese Language Studies, The Education University of Hong Kong, Tai Po, Hong Kong SAR, China; ^2^Faculty of Chinese Language and Culture, Guangdong University of Foreign Studies, Guangzhou, China

**Keywords:** task motivation, task anxiety, Vietnamese university students, L2 writing tasks, different task groups

## Abstract

**Introduction:**

Improving task motivation can reduce anxiety and enhance the efficiency of second language (L2) learning. However, previous research has not determined whether the relationship between task motivation and anxiety is unidirectional facilitation or bidirectional interaction. The reasons for these “relationships” and their impact on L2 learning have not been analysed in depth yet.

**Methods:**

This study investigated the interaction between task motivation and anxiety via qualitative and quantitative research methods with the participation of 229 Vietnamese university students, who were divided into three L2 writing task groups, including the free choice group (FC), the limited choice group (LC), and the no choice group (NC).

**Results:**

The quantitative results show that the higher individuals’ autonomy levels were, the higher their task motivation levels would be. Besides, the high level of task anxiety reduced task motivation among Vietnamese university students and exited other anxiety factors. The qualitative analysis of semi-structured interviews conducted with 32 Vietnamese university students showed that a small number of negative factors might trigger low levels of task anxiety.

**Discussion:**

Nevertheless, the results for participants with different levels of Chinese language proficiency were highly variable. Participants with better cognitive and Chinese language levels regarded task anxiety as an opportunity to practice their Chinese language skills. They were motivated to complete the task, while participants with lower Chinese language levels exhibited low confidence and experienced more challenges when completing the task.

## 1 Introduction

Setting different tasks in the writing activities can improve Chinese as a second language (CSL) learners’ interest in learning and improve their second language(L2) learning capability (Zhai, 2020). Enhancing motivation during task participation can improve learners’ performance and learning effectiveness (Robinson, 2011). *Task motivation* is defined as a set of idiosyncratic motivations or learners’ general motivational dispositions and state motivation, i.e., the performance of a motivated learner on a given task (Jülkunen, 1989). There are many types of motivation concerning L2 learning, among which task motivation is a critical one, which indicates the status quo of motivated learners during task-based activities and their perception and feeling toward the task (Dörnyei, 2002). Promotion in task motivation can lead to more positive and active behaviours, e.g., attitudes towards L2 learning improve significantly with increased task motivation, whereas negative personal emotions, especially anxiety levels, tend to decrease in such situations (Wang and Cheng, 2016; Dörnyei, 2019; Guo et al., 2020).

Anxiety is a sense of stress and fear expressed during L2 learning that is determined by learners’ language abilities, psychosocial phenomena, cognitive factors, and classroom practices (Wang et al., 2021). Different components of anxiety are correlated with learners’ performance, which is related to learners’ language level or language proficiency (Kim and Tracy-Ventura, 2011; Révész, 2011). Take Vietnamese university students as an example; task anxiety can trigger high levels of anxiety, cause the failure of task completion during Chinese language learning, and directly lead to a regression in their task motivation level (Phan, 2011; Trang et al., 2012; Kieu and Liu, 2018). However, some studies suggested that the right amount of anxiety can increase Vietnamese university students’ task motivation and accelerate the completion of L2 learning tasks (Kormos and Wilby, 2020). Autonomy and motivation during tasks are also positively correlated. For example, Vietnamese university students with higher levels of autonomy also have significantly higher levels of task motivation, and their anxiety levels stay relatively stable (Nguyen and Jaspaert, 2021; Vo, 2021). Thus, task anxiety among Vietnamese university students varies under different factors, directly or indirectly affecting task motivation. Task anxiety impacts the learner’s performance during tasks in class negatively, especially in writing (Rahimi and Zhang, 2019). Although the task difficulty level increased, learners could maintain a stable level of task anxiety through writing practice (Wang et al., 2021).

The instruments used in previous studies failed to measure the differences in learners’ task motivation and anxiety during language use, and they did not give sufficient attention to individual variables in the task context. Although these studies analysed internal factors influencing motivation and anxiety under task and the predictive effects of different factors, they did discuss the effects produced by these predictors.

To fill these gaps, this study designed three L2 writing tasks to examine how different writing tasks affect Vietnamese university students’ task motivation and anxiety, the predictive roles of both traits and the relevant factors affecting task motivation and anxiety. Three research questions (RQs) were addressed in the study.

RQ1. How do task motivation and anxiety affect Vietnamese students’ L2 writing under different tasks?

RQ2. What is the relationship between task motivation and anxiety of Vietnamese university students under different L2 writing tasks?

RQ3. What factors attribute to task motivation and anxiety of Vietnamese university students under different L2 writing tasks?

## 2 Literature review

### 2.1 Task motivation and related research in L2 contexts

Task motivation consists of trait motivation, i.e., learners’ general motivational dispositions, and state motivation, i.e., motivation to perform a given task (Kormos and Wilby, 2020). Dörnyei (2009) found that task motivation includes task execution, appraisal, and action control. In empirical research, studying task motivation requires focusing on the dynamics and complexity of other variables (Dörnyei and Kormos, 2000). However, Vietnamese university students possess higher task motivation for reading when compared to other L2 learners. As students’ levels of task motivation increase, their ability to comprehend the learning content and desire to challenge themselves improve correspondingly (Nguyen and Jaspaert, 2021; Vo, 2021). Other researchers have argued that Vietnamese university students’ task motivation varies significantly in their writing under different writing tasks and task selection autonomy levels (Nguyen and Jaspaert, 2021).

Researchers have found that high levels of task motivation promote cognitive and behavioural engagement and learners with higher task motivation tend to improve their language fluency (Kormos and Wilby, 2020) with tasks which were inherently interesting and could motivate learners to invest more effort in choosing their words accurately to complete the corresponding task (Mozgalina, 2015; Kormos and Préfontaine, 2017). Compared with other L2 learners, Vietnamese university students’ task motivation is more likely influenced by language proficiency, interests, communicative purpose, and emotional state (Saito et al., 2018; Nguyen and Jaspaert, 2021; Vo, 2021). Therefore, to understand the mechanisms underlying task motivation in the context of L2 learning, researchers have emphasized learners’ emotional states, especially the emotional changes, such as anxiety, in Vietnamese university students during Chinese learning tasks (Kieu and Liu, 2018; Saito et al., 2018; Jang and Papi, 2021). Research has shown that the level of task motivation of L2 learners is directly affected by anxiety (Philp and Duchesne, 2016). Certain forms of anxiety, such as those stemming from family income, cultural differences, and language environment, can directly reduce task motivation. Conversely, anxieties related to the extent of strategy use and learners’ developmental stage can, to some degree, compensate for a lack of task motivation, thereby enhancing the L2 learners’ motivation levels (Phan, 2011; MacIntyre and Serroul, 2015).

### 2.2 The role of task anxiety

#### 2.2.1 L2 anxiety: facilitative and debilitative?

Anxiety is either a temporary state or a stable trait in L2 learning. The former is transient and exhibits a great deal of fluctuation, whereas the latter, as a trait, is difficult to change (Spielberger et al., 1983). L2 anxiety can be either an instantaneous emotional response to a specific situation or an emotional response to the influence of others in different situations (Jang and Papi, 2021). Furthermore, anxiety resembles certain positive factors, such as competitiveness, which causes students to work harder and helps them overcome learning difficulties (Zhang et al., 2020). Anxiety is an essential factor that affects L2 learning. Firstly, high levels of anxiety cause Vietnamese university students to underestimate the importance of L2 learning, and this phenomenon varies considerably across various levels of Vietnamese universities. Secondly, a moderate level of anxiety can, paradoxically, prove beneficial for these students by boosting their motivation to learn (Trang et al., 2012). Overall, previous research has suggested that anxiety has a positive effect, but more detailed analysis focusing on the factors that influence this positive impact is needed.

#### 2.2.2 The relationship between task motivation and anxiety in L2 contexts

Task anxiety, which refers to a variation in learners’ emotions when they engage in tasks in L2 learning, is strongly correlated with learners’ motivation (Philp and Duchesne, 2016). Learners’ motivation lacks motivational potency when the link between motivation and learners’ emotions, e.g., anxiety, is neglected (MacIntyre and Serroul, 2015). Researchers have categorized learners’ motivations and searched for evidence of a connection between motivational variables and anxiety (Papi and Teimouri, 2014). Firstly, research has shown a positive correlation between the “ought-to L2 self” motivation variable and L2 anxiety, which positively impacts the motivational behavior of L2 learners (Papi, 2010). However, this correlation appears to be specifically significant for prevention-oriented L2 learners. This is due to the notion that anxiety can generate behavior that aligns with the preventative expectations of these learners, thus helping them evade negative consequences (Papi and Teimouri, 2014). Moreover, subsequent studies have discovered that anxiety correlates solely with the “ought-to L2 self” and not with the “ideal L2 self.” It is presumably because anxiety aligns with the preventive motivational orientation of L2 learners. Consequently, it is facilitative by keeping them vigilant of potential adverse outcomes (Teimouri, 2017).

The investigation of Vietnamese university students found that students with higher levels of L2 proficiency showed higher task motivation in their learning sessions, while other affective variables, such as anxiety, work together to facilitate the completion of L2 learning tasks under the influence of task motivation (Vandergrift, 2005).

In addition, Vietnamese university students performed higher task motivation under an L2 learning task that permits higher autonomy of choice (Nguyen and Jaspaert, 2021). However, the more highly motivated learners are, the more likely they are to make errors, and the higher the anxiety levels are (Di Rosa et al., 2021). L2 learning tasks *per se* also impact task anxiety. For example, there are significant differences in anxiety levels across Vietnamese university students based on the learning task (Trang et al., 2012).

Although recent research has focused on task motivation and anxiety, the levels of task motivation and anxiety experienced by L2 learners in different tasks and individual learner characteristics have not been adequately investigated. Although the correlation between task motivation and anxiety is definite (Kormos and Dörnyei, 2004; MacIntyre and Serroul, 2015), whether the correlation is interactive or mutually constraining is not determined. As task anxiety is relevant to mood changes among different learners as well (Philp and Duchesne, 2016), the possibility of making predictions from task motivation to other factors and vice versa must be discussed as soon as the factors involved in participants’ task anxiety are clarified. As for the research method, most previous studies drew on quantitative approaches regarding the factors that influence task motivation and anxiety. However, questionnaires heavily rely on items prepared by researchers that cannot fully explain the characteristics of motivation and anxiety in language learning tasks and fail to obtain the participants’ views. Therefore, a combination of qualitative research, such as semi-structured interviews and quantitative research, is needed to provide a more in-depth and comprehensive analysis of these subjects.

## 3 Methodology

### 3.1 Participants

Participants in this study were Chinese language students in their junior or senior year at a university in Vietnam. All participants’ L1 was Vietnamese, and L2 was Chinese. 235 participants were included, with ages ranging between 18 and 24 years (one participant was aged 25 or older). Data collection of the study lasted from December 5^th^ to December 14^th^, 2021. Six participants who did not complete the writing task and questionnaire were excluded. The final study included 229 participants (male = 14, female = 215). The gender disparity of the study resulted from the unbalanced proportion of male and female students majoring in Chinese at the university.

The participants need to meet two criteria to participate in the study. First, they needed to have taken the *Chinese writing course*, which aimed to train the students in Chinese writing for at least 2 years. Second, they had to obtain a passing score for the Chinese writing course.

### 3.2 Instrument and the material

#### 3.2.1 Questionnaire

The questionnaire used in this study, which focused on investigating task motivation and anxiety among Vietnamese university students, consisted of two parts. The first part introduced the main content of this study and inquired into participants’ basic information, such as gender, group affiliation, time spent studying Chinese, and other primary content. The second part of the questionnaire investigated task motivation and anxiety during L2 writing. It was adapted from *the task motivation and task anxiety questionnaire item* (Wang et al., 2021), which can accurately reflect the motivation and anxiety levels of L2 learners during the task and reveal the patterns of changes in both, with high reliabilities. 952, and 0.941 in task motivation and anxiety, respectively. This part of the questionnaire was divided into two sections: Section 1 consisted of 33 items involving the task motivation and was divided into three subsections. The first subsection focused on positive attitudes towards the task, indicating the learner’s positive attitude towards the task (e.g., *this activity was interesting*) and including items 1, 2, 7, 9, 15, 19, 24, 25, 26, and 32. The second subsection emphasized negative attitudes towards the task, which illustrated the learner’s negative attitude towards the task (e.g., *I did not like doing this oral task*) and included items 3, 8, 10, 11, 12, 13, 14, 16, 17, 18, 22, 23, 31, and 33. The third subsection, which contained items 4, 5, 6, 17, 18, 20, 21, 27, 28, 29, and 30, highlighted the task as a diagnostic tool and demonstrated the role played by the task activity (e.g., *this task was useful because it helped me recognize my weaknesses*).

Section 2 included the task anxiety questionnaire with 41 items and was divided into three subsections. The first subsection focused on language-related anxiety, indicating language difficulties encountered by the learner while completing the task (e.g., *I felt nervous when I could not think of a word to describe something*) with items 34, 40, 41, 42, 43, 44, 47, 53, 54, 55, 56, 57, 60, 62, 63, 64, 65, 67, 68, 71, and 73. The second subsection employed items 35, 36, 37, 38, 39, 61, 66, 69, 70, 72, and 74 to emphasize anxiety relief, indicating the features and aspects of the task that made participants feel less anxious (e.g., *I did not feel nervous because the atmosphere was relaxing*). The third subsection highlighted setting related anxiety and physiological symptoms with items 45, 46, 48, 49, 50, 51, 52, 58, and 59, which elaborated aspects of the anxiety-inducing task setting and the participant’s anxiety-induced physiological symptoms (e.g., *I felt nervous because of the audio and video recording*).

The questionnaire was designed bilingual to help the participants understand each item thoroughly and reduce the duration of the questionnaire. The Chinese version was cross-checked by the three authors of this study, while the Vietnamese version was translated and revised by a Chinese language teacher from a Vietnamese university (the teacher). A 5-point Likert Scale was used to score both parts of the questionnaire, with responses including “1” = “*never*,” “2” = “*rarely*,” “3” = “*sometimes*,” and “4” = “*often*,” and “5” = “*always*.” It took10-15 min to complete the questionnaire.

#### 3.2.2 Semi-structured interviews

After completing the questionnaire, 32 participants in this study were randomly selected for a semi-structured interview via Zoom independently, which took 15–25 min. The interview questions were drawn from Gregersen et al. (2014) and MacIntyre and Serroul (2015) and were intended to help us obtain a comprehensive understanding of the factors that influence and limit Vietnamese university students’ task motivation and anxiety during writing tasks. Nineteen questions were asked during these interviews, and the first three focused on basic information, i.e., name, group, and writing topic. The other 16 questions inquired into task motivation and anxiety, e.g., “*Do you feel anxious before the task starts?*” or “*When you feel anxious, are you more motivated or less willing to complete the task?*” This study encouraged Vietnamese university students to express themselves freely regardless topics to obtain accurate data. Both the interviewer and the interviewees spoke Chinese during the interviews, and all the interviews were recorded by Zoom and ultimately converted to the MP4 file format. The audio files verbatim were transcribed into Word documents by the first author and checked by the second author before being importing into NVivo 12 for analysis. NVivo is a robust software tool designed for qualitative analysis. It’s capable of coding a wide range of data types, such as text, pictures, audio, and video. This software assists in identifying the factors that impact task motivation and anxiety within semi-structured interview data. Moreover, it facilitates the ensuring exploration, generalization, and summarization of the underlying causes for these factors (Welsh, 2002).

#### 3.2.3 Task material

Each participant was required to start the task by reading the task material, which consisted of 764 words, with a time limit of 10–15 min. We used two passages from “*Developing Chinese - Writing Chinese at Middle Level*” (Luo, 2006). The first paragraph was the “Learning Scheme” that discussed a student’s Chinese language learning arrangements and future Chinese language learning plans. The other paragraph was the “*Travel Scheme*,” which narrated the story of a Chinese language student studying in China who used his holidays to travel. According to the assessment of the teacher, both passages comprised opening sentences, transitional sentences, and concluding sentences, highlighting the primary content of L2 writing, and meeting the practical writing needs of junior and senior students studying the Chinese language.

### 3.3 Procedures

Step I: This study was conducted concerning the principle of random sampling to ensure each participant could attend the writing task with equal opportunities. The participants, who were required to complete a writing task after reading, were randomly divided into three groups: the free choice (FC) group (*n* = 89), the limited choice (LC) group (84), and the no-choice (NC) group (*n* = 84). The same reading material but different writing tasks were assigned to the three groups. The duration (a maximum of 45 min) and the word limitation (a maximum of 500 Chinese words) for the three groups were the same. The FC group was asked to write an essay related to the task material after reading without any restrictions. The LC group was required to choose one of the three assigned writing topics for writing, i.e., “我的_____计划 (My _____ plan),” “_____的理想 (____’s dream),” and “___的愿望 (_____’s wish).” The NC group was demanded to write on a fixed topic, “我的_____计划 (My _____ plan).”

All the essays were graded separately by the teacher and the first author based on the scoring standard of Chinese writing at the university, from three aspects, including overall impression (topic selection and paragraphing), content (beginning, transition, and ending), and language (correctness and diversity), with a total score of 10. The final score of an essay is the average of the two scores. In addition, according to the teacher’s suggestion, the second author and the third author will score the compositions with a discrepancy of over 1 point separately. Finally, the lowest and the highest scores will be removed, and the average score of the two intermediate scores will be taken as the final score.

Step II: After completing the writing task, each participant was asked to complete a questionnaire in a Google Form.

Step III: 32 participants were randomly selected to participate in a one-on-one semi-structured interview via Zoom.

To ensure the reliability and accuracy of the data, a pilot study was conducted before the survey was administered. The pilot study was launched on December 5th, 2021, with 36 participants (male =1, female = 35). One male and two female participants were included in the interview. Based on the pilot study, the time allotted to the writing task was increased from 35 to 45 min after discussion and deliberation with the teacher. Besides, repeated questions were removed, and the sequence of items was rearranged. Finally, the questionnaire was formally distributed to all participants.

### 3.4 Data analysis

First, the results of the writing task were analysed by using SPSS 27. Descriptive statistics was employed to investigate the performance of Vietnamese university students in different tasks (RQ1). Second, the exploratory factor analysis (EFA) and confirmatory factor analysis (CFA) were adopted to analyse the data obtained from the questionnaire to ensure the reliability of the internal structure of the questionnaire. Third, the correlations between different task motivational variables and task anxiety were examined by using structural equation modelling via AMOS 27 (RQ2). Finally, two of the authors coded the data collected from the interviews independently by using NVivo 12 to ensure a high degree of consistency between the two sets of codes. The factors influencing task motivation and anxiety (RQ3) were analysed based on this coding.

#### 3.4.1 Data processing for the questionnaire

The reliability of the questionnaire was examined by using SPSS 27 to determine Cronbach’s alpha coefficient. The reliability for task motivation and anxiety were 0.952 and 0.941, respectively, greater than 0.9. This study tested the questionnaire via factor analysis to extract relevant questions. It was necessary to test the suitability of the questionnaire for factor analysis. The Kaiser-Meyer-Olkin (KMO) measure of sampling adequacy and Bartlett’s test of sphericity were used. The results showed that the KMO values for task motivation and anxiety were 0.949 and 0.908, respectively, indicating a high degree of significance at *p* < 0.001.

Based on the previous step, EFA and CFA were combined to determine the internal factor structures of task motivation and anxiety. Regarding EFA, the principal components analysis function of SPSS 27 was used as the extraction method, and the rotation method of varimax with Kaiser normalization was employed (Kaiser, 1974). Items that exhibited higher factor loadings, i.e., greater than 0.5, were retained. Therefore, 16 and 17 items were retained for task motivation and anxiety, respectively. According to previous subscale standards (Azkarai and Kopinska, 2020; Wang et al., 2021), task motivation included achievement motivation (AM), the expectancy of success (ES), and subjective task value (STV). The three factors explained 62.048% of the total variance (see Table 1). Task anxiety, which included state anxiety (SA), individual performance (IP), task difficulty (TD), and language accuracy (LA), explained 59.534% of the total variance (see Table 2). Regarding CFA, analysis of the extracted factors using AMOS 27 showed that both models reached acceptable levels for all indicators, with GFI, CFI, TLI, and IFI greater than 0.8, RMSEA ≤0.08, and CMIN/DF < 3. These results indicated that the models were overall better, and it was appropriate to proceed to the subsequent step of the analysis.

**Table 1:** The results of EFA for task motivation.

	Item	AM	ES	STV
Task motivation	1	0.723		
	2	0.707		
	4	0.738		
	6	0.803		
	7	0.738		
	11	0.694		
	12	0.76		
	27	0.789		
	28	0.792		
	29	0.76		
	16		0.748	
	17		0.707	
	32		0.781	
	3			0.846
	10			0.823
	33			0.706
	Eigenvalue	6.453	2.024	1.451
	Variance explained in percentage (Total 62.048%)	40.331%	12.649%	9.906%

Achievement motivation = AM, the expectancy of success = ES, subjective task value = STV.

**Table 2:** The results of EFA for task anxiety.

	Item	SA	IP	TD	LA
Task anxiety	36	0.762			
	37	0.862			
	38	0.834			
	40	0.805			
	42	0.741			
	34		0.603		
	46		0.675		
	47		0.651		
	51		0.668		
	61		0.663		
	66		0.609		
	35			0.7	
	50			0.627	
	74			0.758	
	63				0.805
	64				0.867
	65				0.572
	Eigenvalue	5.621	2.767	2.325	1.194
	Variance explained in percentage (Total 59.534%)	28.103%	13.834%	11.626%	5.97%

State anxiety = SA, individual performance = IP, task difficulty = TD, language accuracy = LA.

#### 3.4.2 Data processing for the semi-structured interviews

Based on previous studies (Mozgalina, 2015; Guo et al., 2020; Wang et al., 2021), a Chinese language teacher from a Vietnamese university and the first author who is experienced in coding schemes were responsible for coding the data. The coding schemes was finalized after several discussions and modifications by the two individuals involved. To ensure uniformity in coding, 30% of the coding data for this study were randomly selected and recoded coded by another researcher with coding experience. NVivo 12 was used to calculate the Kappa coefficient and the level of agreement (see Table 3).

**Table 3:** Intercoder reliability.

Themes			Kappa coefficient	Agreement (%)
Motivation	Internal factors	Interests and hobbies	0.9646	99.81
		Perceived efforts	0.7556	88.27
		Influence on future learning	0.8596	98.32
		Self-confidence	0.6434	92.03
		Language proficiency (Chinese)	0.6733	94.75
	External factors	Task characteristics	0.848	95.5
		Task design	0.5632	87.97
		Task difficulty	0.9514	99.81
		Language features (Chinese)	0.644	87.96
Anxiety	Task factors	Task arrangement	0.5053	85.69
		Task difficulty	0.5188	90.99
		Task familiarity	0.5923	95.65
	Learner factors	Cognitive level	0.9721	99.83
		Language competence (Chinese)	0.5115	78.5
		Personal response	0.5681	89.91
		Future plans	0.8593	99.64
	Other factors	Curriculum arrangement (Chinese)	0.4784	97.59
		COVID-19	0.5459	98.88

As Table 3 indicates, all Kappa coefficients exceeded 0.4 and the overall level of agreement was over 70%, with individual Kappa coefficient values surpassing 0.9. These values fall within the acceptable range, demonstrating high reliability and validity (Kaiser, 1974). These results indicated that the coding conducted by the two researchers is highly consistent.

## 4 Results

### 4.1 Measures and scores of Vietnamese university students under different tasks

First, descriptive statistics was employed to analyse the performance of Vietnamese university students by the descriptive statistics of the questionnaire (see Table 4).

**Table 4:** Description of results.

		Mean	Std. deviation	Std. error	95% Confidence interval for mean	
					Lower bound	Upper bound
Achievement motivation	FC	4.3135	0.55025	0.05833	4.1976	4.4294
	LC	4.256	0.61612	0.06722	4.1222	4.3897
	NC	4.2696	0.66358	0.08867	4.0919	4.4473
Expectancy of success	FC	3.9813	0.69333	0.07349	3.8352	4.1273
	LC	3.9008	0.74226	0.08099	3.7397	4.0436
	NC	3.8392	0.76275	0.10193	3.635	4.0436
Subjective task value	FC	2.0824	1.0302	0.1092	1.8654	2.2994
	LC	1.9603	0.90129	0.09834	1.7647	2.1559
	NC	2.1429	1.07282	0.14336	1.8556	2.4302
State anxiety	FC	3.7618	0.84685	0.08977	3.5834	3.9402
	LC	3.769	0.89523	0.09768	3.5748	3.9633
	NC	3.6893	0.77311	0.10331	3.4822	3.8963
Individual performance	FC	2.7022	0.7985	0.08464	2.534	2.8705
	LC	2.619	0.7737	0.08442	2.4511	2.787
	NC	2.6674	0.88942	0.11885	2.4292	2.9056
Task difficulty	FC	3.4972	0.77285	0.08192	3.3344	3.66
	LC	3.494	0.84131	0.09179	3.3115	3.6766
	NC	3.6205	0.7119	0.09513	3.4299	3.8112
Language accuracy	FC	3.8015	0.82835	0.0878	3.627	3.976
	LC	3.7579	0.7733	0.08437	3.5901	3.9258
	NC	3.6964	0.81965	0.10953	3.4769	3.9159

According to the results shown in Table 4, the means of the three task motivation factors were similar across the different groups of Vietnamese university students, but in terms of standard deviation and standard error, FC was the smallest, LC was in the middle, and NC was the largest. Differences among participants increased with increasing task demands and decreasing selectivity, i.e., FC to LC to NC. Specifically, FC exhibited the highest mean for task motivation for the different groups. The 95% confidence interval range of the mean for this group was higher than those of the other two groups, i.e., FC demonstrated the highest levels of task motivation. LC exhibited different results for task motivation and anxiety. The higher standard deviation and standard error found for NC concerning task anxiety compared to the other two groups indicated that students in this group had higher anxiety levels regarding the writing task and highlighted that this group featured more considerable differences among students. These results suggested that as the task demands increased, participants’ motivation to perform the task tended to decrease, whereas task anxiety increased.

### 4.2 Predictive effects among factors of task motivation and anxiety

**Figure 1:**
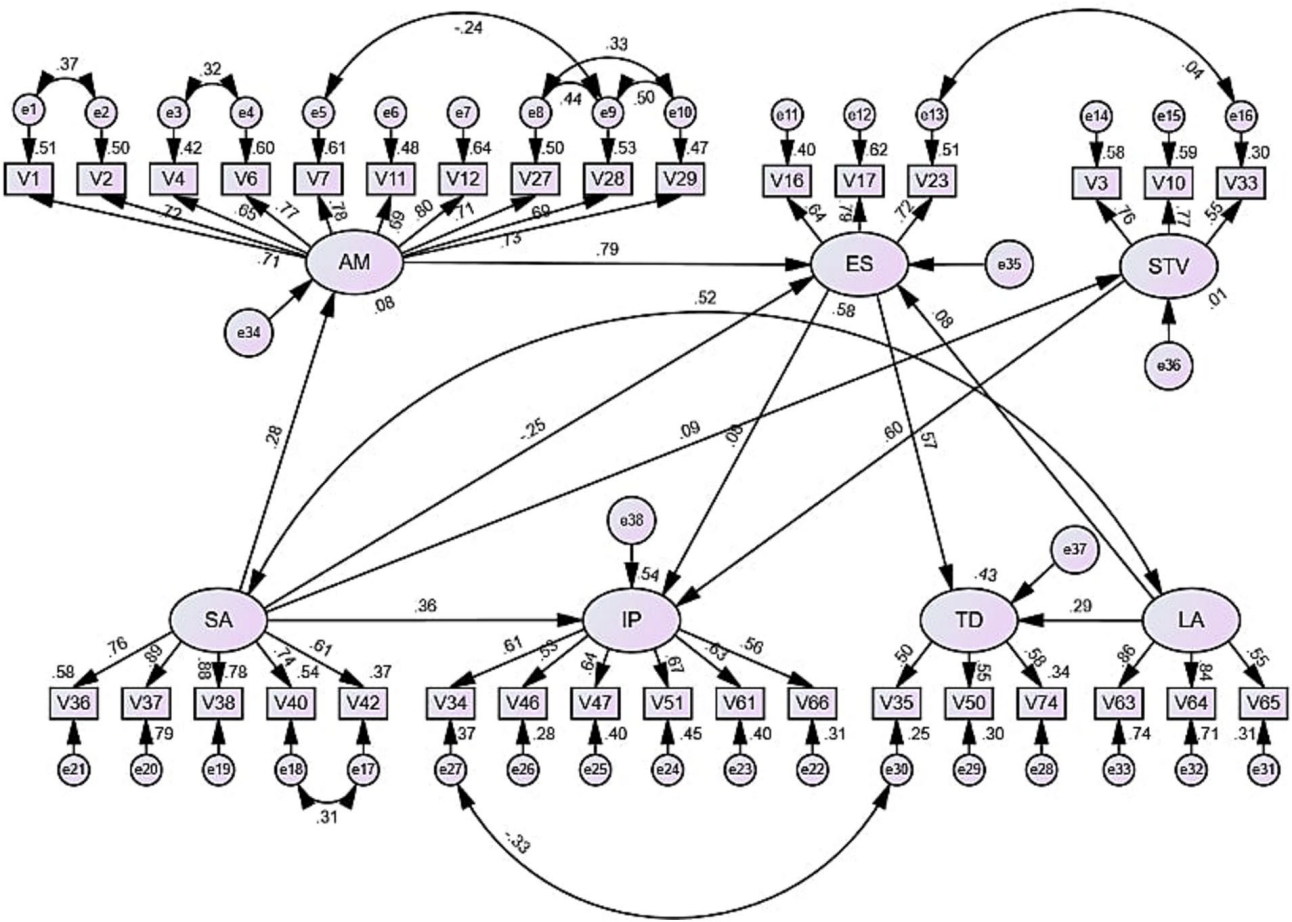
The relationship between task motivation and anxiety. AM = achievement motivation, ES = expectancy of success, STV=subjective task value, SA = state anxiety, IP = individual performance, TD = task difficulty, LA = language accuracy.

A structural equation model was constructed by AMOS 27 to test the predictive effects among factors of task motivation and anxiety. After several adjustments, the model had relatively high indicators, with GFI, CFI, TLI, and IFI > 0.8, RMSEA ≤ 0.08, and CMIN/DF < 3. The regression coefficients of −0.25 for SA to ES and 0.79 for AM to ES reached the significance level. SA and AM explained 57.6% of the total variance in ES. In addition, the regression coefficients for SA and STV to individual performance (IP) were significant (*p* < 0.001), explaining a total of 54% of the variance in IP (see Figure 1; Tables 5, 6).

**Table 5:** Summaries the total, direct, and indirect effects.

Items	Direct effects	Indirect effects	Total effects
SA on ES	−0.248	0.223	−0.025
AM on ES	0.788	0	0.788
SA on IP	0.375	0.053	0.41
STV on IP	0.605	0	0.605

AM = achievement motivation, ES = expectancy of success, STV = subjective task value, SA = state anxiety, IP = individual performance.

**Table 6:** Standardized regression weights.

Items	Estimate	P Label
SA on AM	0.283	***
SA on ES	−0.248	0.003
SA on STV	0.090	0.256
AM on ES	0.788	***
LA on ES	0.082	0.297
SA on IP	0.357	***
ES on IP	0.081	0.231
STV on IP	0.605	***
LA on TD	0.287	0.003
ES on TD	0.571	***

Achievement motivation = AM, expectancy of success = ES, subjective task value = STV, state anxiety = SA, individual performance *=* IP, *** = *p*-value < 0.001.

The results of the data analysis reveal that SA affected ES through AM, both directly and indirectly. The direct effect was −0.248, and the indirect impact was 0.223. Both effects were similar in value. These results reflected a negative correlation between SA and ES. In addition, there was both a direct and an indirect effect between SA and IP. The direct impact was 0.375, and the indirect impact was 0.053. Although both results were positive, the direct effect was much more significant than the indirect one. ES had a direct effect on TD, with an impact of 0.57. This suggests that as ES increases, there is a tendency for TD to grow well. More importantly, there was a direct effect between STV and IP of 0.605, a higher value than the direct effect between SA and IP. Overall, task anxiety and motivation had a significant direct impact on individual performance. Attention should be paid to task motivation’s effects on task anxiety and the correlations between the factor structures inherent in task anxiety.

### 4.3 The coding categories of task motivation and anxiety

#### 4.3.1 Categorization of task motivation

In line with previous work (Mozgalina, 2015; Wang et al., 2021), task motivation was divided into internal and external factors. Internal factors included five aspects: interests and hobbies, perceived efforts, influence on future learning, self-confidence, and personal Chinese language proficiency, while external factors included four aspects: task characteristics, task design, task difficulty, and language features (Chinese; see Appendix A).

Internal and external factors were similar in number and frequency, indicating that both factors had identical effects on task motivation. In this context, 27 Vietnamese university students mentioned perceived effort 30 times, which was positive. This result showed that most Vietnamese students were well prepared and motivated to complete the task. Although the number and frequency of mentions were high for personal Chinese language proficiency, positive and negative content was present in specific responses. The negative content mainly focused on a lack of mastery of Chinese vocabulary, grammar, and other content. Lower-level students made errors in word usage and experienced grammatical confusion, which affected their motivation to complete the task. Interests and preferences were mainly related to personal preferences for Chinese learning. Sixteen participants mentioned this factor, while its frequency was 65, which was positive and higher than other factors. It was clear that interests and hobbies were essential factors influencing participants’ motivation to complete this task. As participants’ interest increases, their task motivation also increases.

Twenty-eight external factors focused on task design. Task design was mainly concerned with the content of this writing task, including learners’ perceptions of the task and attitudes. The frequency of mentions of this factor was 54 times higher than other external factors. However, most participants’ attitudes were neutral or negative concerning this thematic content. For example, participants’ responses included “*I would like to increase the task duration” and “Reduce the content of this task*.” This phenomenon identified task design as a negative factor directly affecting Vietnamese university students’ task motivation.

Regarding the difficulty and characteristics of the task, some participants showed a willingness to “*reduce the task’s difficulty*.” However, they insisted on completing the task and noted that they would “*continue to learn Chinese in the future*.” In terms of the characteristics of the Chinese language, 9 participants mentioned this factor with a frequency of 10 mentions. Regarding thematic content, most participants experienced difficulty learning Chinese, such as insufficient vocabulary and confusing grammar. These factors could directly affect their interest in participating in this task and future Chinese language learning. Overall, despite negative factors such as Chinese language proficiency, the 229 participants’ motivation to complete the task showed positive results due to internal and external factors.

#### 4.3.2 Categorization of task anxiety

Based on the previous categorization (Guo et al., 2020; Wang et al., 2021), this study summarized task anxiety factors into three categories: task factors, learner factors, and other factors. Task factors comprise task arrangement, difficulty selection, and task familiarity. Learner factors included cognitive level, language competence, personal response, and plans. Other factors included curriculum arrangement (Chinese) and COVID-19 (see Appendix B).

Among task factors, the task arrangement was mentioned most frequently, with 29 individuals mentioning this factor for a total of 69 mentions. By analysing the content themes, some participants expressed significant anxiety and nervousness levels at the beginning of the task because they were not informed of the task procedures in advance, a persistent finding. Combined with task familiarity, most participants had not previously participated in a similar writing task, which resulted in an overall high level of task anxiety among Vietnamese university students. Regarding learner factors, the highest frequency of mentions was for personal response at 72, which included themes on participants’ inability to complete the writing task on time. In addition, participants had deficiencies in their Chinese language proficiency for cognitive level and language competence.

In contrast, other participants expressed high levels of tension and anxiety due to the task’s difficulty, such as “*feeling anxious when encountering difficulties*.” This result indicated that the difficulty of this writing task caused lower-level Chinese learners to exhibit higher levels of anxiety during the task. Only four participants mentioned plans, but the subject matter of these mentions involved the need to improve Chinese language learning and reduce language tension and stress. Other factors were also relevant, such as the influence of curriculum arrangement (Chinese) and COVID-19. However, the thematic content was primarily positive for participants’ desires to alter the Chinese language curriculum, adapt the mode of Chinese language teaching, and adjust the mode of Chinese language teaching to permit active participation in offline classroom learning activities. These results reflected that some participants were eager to improve the current situation of Chinese language learning.

In general, Vietnamese university students experience high levels of task anxiety due to issues such as the difficulty of the task and their deficiencies in Chinese language proficiency. Nevertheless, they continued learning Chinese and hoped to improve their weaknesses and enhance their Chinese language proficiency in various ways.

## 5 Discussion

### 5.1 Task motivation and anxiety of Vietnamese university students under different writing tasks

Based on the adjusted and modified model, the differences between Vietnamese university students’ task motivation and anxiety under the three L2 writing tasks are discussed (RQ1). The results showed that the choice of various tasks did not affect task motivation and anxiety, as the results of previous research (Mozgalina, 2015). In comparison, participants in the remaining two groups showed a significant decline in task motivation as task selectivity decreased. In L2 learning, a high degree of autonomy is a crucial element that influences motivation (Mozgalina, 2015). Participants in FC had higher levels of autonomous choice. As a result, they showed higher motivation levels for language learning, which led to active participation in language learning activities and better language learning outcomes. The study found that the NC group exhibited higher anxiety levels than the LC group and was similar to the FC group regarding task anxiety. Since participants in the NC group had no options, they were required to complete the corresponding tasks according to the assigned instructions. The task could be more stressful and overwhelming for the less motivated participants, who demonstrated more fear and even withdrawal behaviour. For participants with lower language proficiency, the lack of autonomous selection for the task could also lead to difficulties in vocabulary and grammar. This result was related to task selectivity (MacIntyre and Serroul, 2015; Guo et al., 2020). When participants were unable to select the L2 writing task, their motivation levels decreased to some degree, thus affecting the successful completion of the task.

### 5.2 Relationships between task motivation and anxiety

The structural equation modeling analysis results indicated a high predictive effect of task motivation on anxiety (RQ2). SA affected IP directly and indirectly through STV, and both direct and indirect effects were positive. This indicated that some task anxiety factors indirectly affect other task anxiety factors through task motivation. The direct effect between ES and AM was positive, whereas the direct effect between ES and SA was negative. Previous research has suggested that SA represents a learner’s anxiety during a task related to the specific task in which the learner is engaged and can be influenced by or interfere with different types of tasks (Byron and Khazanchi, 2011). In contrast, IP showed how anxiety affects participants’ performance on the task. STV and ES, as two task motivation factors, serves as an essential indicator to assess the interest, effort, and successful value created by participants in the task (Wang et al., 2021). Therefore, Vietnamese university students’ task motivation was affected by their anxiety level and would indirectly affect their performance in the writing task. Excessive anxiety would directly cause a decrease in task motivation and lead to less satisfied results in Vietnamese university students’ writing.

This result suggests a strong correlation among factors within task motivation. Firstly, task anxiety factors indirectly influence other task anxiety factors through task motivation. Under the influence of external and internal factors, growth in one anxiety factor directly or indirectly affects the improvement of other factors. This finding supports the previous theory of task motivation, which emphasizes the importance of considering the dynamics of different variables (Dörnyei and Kormos, 2000). Furthermore, it adds a new dimension to the internal complexity of task motivation theory. Specifically, it reveals the role of different task motivation factors in relation to anxiety in L2 learning tasks.

Secondly, during writing, the Vietnamese university students experienced anxiety with some degree of variation among different groups. As Vietnamese university students were not informed of the writing tasks in advance, they were anxious when receiving the writing task, which in turn affected their task motivation, causing Vietnamese university students to experience more negative states, thereby affecting their final writing effectiveness. These phenomena corroborate the theory of L2 anxiety, which posits that the temporary emotional responses can occur in task-specific situations (Jang and Papi, 2021). Notably, the extent of this response showed considerable variations among the different groups of Vietnamese university students.

### 5.3 Factors attributed to task motivation and anxiety

#### 5.3.1 Factors affecting task motivation

The data drawn from the semi-structured interviews revealed both internal and external factors that influence task motivation (RQ3). Among internal factors, language proficiency, perceived effort, and interest were crucial influences on participants’ motivation to complete the task. When participants had a high level of interest in the task, they showed a high degree of task motivation, demonstrated better vocabulary and grammar, and were more willing to put in the effort to complete the task. This result also suggested a high positive correlation between task motivation and effort level. When participants exhibited higher motivation levels, they were more motivated to put effort into the task to obtain better results. In addition, task characteristics and design were the main elements found among external factors. In this context, task design emerged as a partially negative factor, with participants desiring to increase the time allotted to tasks and decrease the task difficulty. However, some participants felt that the number and difficulty of tasks needed to increase to practice their Chinese language skills. Therefore, L2 learning tasks should feature more novel elements and be varied and challenging (Dörnyei and Ushioda, 2011). However, participants with low Chinese language proficiency and insufficient self-confidence wished to make existing tasks less complex and be given a longer time to complete these tasks.

In studying the language features (Chinese), a small number of participants could not accurately distinguish the meanings of similar words. However, these participants still expressed a higher motivation for task participation and tended to participate in more Chinese learning tasks. In other words, a deficiency in proficiency level did not affect participants’ motivation to participate in the task. Overall, when examined by combining internal and external factors, 32 participants exhibited strong motivation to complete the task. Individual negative factors did not affect participants’ task motivation but instead motivated them to display higher levels of motivation and initiative. This outcome proposes that while detrimental factors like vocabulary proficiency may impact Vietnamese university students’ L2 learning, they do not decrease their L2 motivation. This discovery fortifies earlier research on task motivation theory, which asserts that task motivation theory, a complex system, is susceptible to alterations engendered by external factors. However, it is imperative to note that these changes do not invariably carry negative implications (MacIntyre and Serroul, 2015).

#### 5.3.2 Factors affecting task anxiety

This study also discussed three factors that influence task anxiety (RQ3). First, since most participants had not been involved in similar tasks, they were more likely to become nervous and overwhelmed and took extra time to familiarize themselves with the task process. Participants experienced anxiety during the task caused by factors such as language ability and unexpected events (Gregersen et al., 2014). Subsequently, several participants performed the writing task on time. However, some participants “*felt that the task was difficult and that I was nervous all the time*” expressed a high level of anxiety and were unable to complete all tasks within the allotted time.

Conversely, anxiety can also increase participants’ concentration on the task, rapidly enhance participants’ attention to the task, and improve their level of motivation. This process strongly correlates with participants’ language ability, academic performance, and competence (Strack et al., 2017). Therefore, for participants with better Chinese language ability, the negative effects of task anxiety might be only temporary. Such individuals were able to adapt to the task in time and regarded anxiety as an essential factor in enhancing motivation. This phenomenon is mirrored in the theory of anxiety in L2 learning. According to this theory, anxiety, as an emotional reaction to different situations, can have both positive and negative effects (facilitative and debilitative) on different L2 learners (Jang and Papi, 2021). It suggests that anxiety could potentially function as a theory of enhanced motivation, fostering initiative and motivation in L2 learners.

16 participants in this study showed anxiety at the cognitive level. Anxiety at the cognitive level may result from negative emotions and increase with the difficulty level of the task (Strack et al., 2017). It can be assumed that some participants eventually experienced anxiety since their negative emotions affected their cognitive level. In addition, under the influence of COVID-19, participants were dissatisfied with the current structure and arrangement of the Chinese language course. They expressed the following desire: “*I wish I could return to the classroom in time to study after the epidemic is over*.” In short, although certain factors might cause anxiety, participants were still eager to enhance their Chinese language learning and improve their Chinese level.

## 6 Conclusion

By investigating 229 Vietnamese university students, this study found that students exhibited higher levels of task motivation than anxiety in different writing tasks. When students had higher levels of autonomous choice (FC), their task motivation was high. For students with a lower level of choice (NC), their levels of task anxiety were significantly higher than those of the other two groups. Second, there was a link between task motivation and anxiety. Task anxiety rose when students determined to complete the task, which directly affected task motivation and subsequently accelerated task anxiety through various factors of task motivation. Third, although some factors were identified to be negative, they did not affect motivation levels. Conversely, they promoted students’ task motivation. Students with higher levels of Chinese language proficiency or a higher level of cognition regarded anxiety as a driving force that could facilitate their Chinese language learning. Correspondingly, they were more motivated and took the initiative in completing the writing task. In general, the motivation of Vietnamese university students to learn Chinese can be significantly enhanced through the accomplishment of the L2 writing task, especially for students with higher levels of Chinese language proficiency. However, for students with deficiencies in Chinese language learning, unfamiliar L2 writing tasks might impact their self-confidence negatively and cause them to experience more incredible difficulties.

Measuring CSL learners’ variability under different writing tasks can clarify the relationship between task motivation and anxiety in CSL and the influencing factors based on a clear view of L2 learners’ task motivation and anxiety levels, which provide a reference for future research on L2 writing. In addition, the study assisted the researchers in identifying the variation rule for task motivation and anxiety in CSL learners, which benefits the L2 writing research and pedagogical understanding of L2 writing.

However, the study has some limitations, too. For example, if there are differences in task motivation and anxiety between participants in different task groups, do these groups correlate or interact with each other? In addition, are there other variables that mediate the relationship between task motivation and anxiety? These issues need to be studied further by future research.

## Data availability statement

The raw data supporting the conclusions of this article will be made available by the authors, without undue reservation.

## Ethics statement

This study was approved by the Human Research Ethics Program at the Education University of Hong Kong.

## Author contributions

CW: research design, data collection and analysis, and research implementation. SZ and HZ: data analysis. All authors contributed to the article and approved the submitted version.
