# Supplementary material for: Interaction between CSL students’ motivation and anxiety under different L2 writing tasks: evidence from Vietnamese university students
Source: Front Psychol. 2023 Nov 23;14:1230498. doi: 10.3389/fpsyg.2023.1230498 (PMC10701526; doi:10.3389/fpsyg.2023.1230498)
Supplement: Supplementary file 1 [file Table_1.docx]

Supplementary Material

Interaction between CSL students’ motivation and anxiety under different L2 writing tasks: Evidence from Vietnamese university students

**Cong Wang^*^, Sida Zhu, Haijing Zhang**

*** Correspondence:** Cong Wang: wangc@eduhk.hk

# Supplementary Data

**Appendix A.** **Factors affecting task motivation.**

| Themes | | Details of theme | N | F |
| --- | --- | --- | --- | --- |
| Internal factors | Interests and hobbies | 1. I like writing very much.  2. I really enjoy writing in Chinese.  3. I’m interested in writing tasks.  4. This task is fascinating to me. | 16 | 65 |
|  | Perceived efforts | 1. For Chinese writing, I will prepare carefully.  2. To better complete this task, I reviewed a lot of Chinese vocabulary a few days ago. | 27 | 30 |
|  | Influence on future learning | 1. I need to learn Chinese seriously to study in China in the future.  2. This task will help me to improve my Chinese language skills. | 5 | 7 |
|  | Self-confidence | 1. I have/do not have the confidence to complete this assignment. | 10 | 14 |
|  | Language proficiency (Chinese) | 1. I can complete this task successfully, because I am good at learning vocabulary.  2. I did very well in vocabulary and grammar.  3. I am not a good writer and make mistakes in my use of vocabulary and grammar.  4. I have a lot of problems with vocabulary learning.  5. I often get confused with Chinese grammar. | 26 | 53 |
| External factor | Task characteristics | 1. This task was unique and I really enjoyed it.  2. Through taking part in this task, I learnt about my shortcomings in Chinese language learning.  3. This task makes me think I need to study Chinese seriously in the future. | 26 | 50 |
|  | Task design | 1. I really enjoyed this assignment, which included multiple sessions.  2. I thought the task was well/badly organised.  3. It was a bit short this time, I couldn’t finish it and was a bit tempted to give up.  4. It would have been nice if there could have been more/less content in this task.  5. I hope that such tasks will be arranged for Chinese writing classes in the future. | 28 | 54 |
|  | Task difficulty | 1. The task was easy for me, and I could have done it easily.  2. The task was a bit difficult for me and I did not do well.  3. I wish the difficulty of the task could be reduced/increased.  4. A more/less difficult task would increase my interest in learning Chinese. | 16 | 25 |
|  | Language features (Chinese) | 1. Chinese is too difficult to learn.  2. Chinese vocabulary is difficult, and I can’t distinguish the meaning of some vocabularies.  3. Chinese grammar is difficult, and I often use it incorrectly.  4. I often make mistakes in vocabulary and grammar, which leads to my interest in learning Chinese becoming low.  5. Although Chinese is difficult, I will study it seriously. | 9 | 10 |

*Note*: N=Number, F= Frequency.

**Appendix B. Factors affecting task anxiety.**

| Themes | | Details of theme | N | F |
| --- | --- | --- | --- | --- |
| Task factors | Task arrangement | 1. The schedule of tasks this time was a bit unreasonable, and I was not able to prepare in advance.  2. I wish we could be told in advance about the next assignment. | 29 | 69 |
|  | Difficulty selection | 1. The task is a bit difficult this time, so I will feel anxious.  2. The task is very difficult for me this time.  3. the higher the level of difficulty, the higher my anxiety level.  4. I would like future tasks to be easier. | 16 | 16 |
|  | Task familiarity | 1. I have not done a task like this before.  2. The unfamiliarity of the task made me feel anxious and nervous.  3. The first time I did such a task, I was particularly nervous at the beginning.  4. After I understood the task, I was less nervous.  5. It took me a long time to get familiar with the task. | 12 | 25 |
| Learner factors | Cognitive level | 1. When I write in Chinese, I get a bit nervous and anxious.  2. I feel anxious when I encounter difficult problems during the task.  3. I am afraid of writing, and I feel nervous and anxious about the task. | 16 | 22 |
|  | Language competence (Chinese) | 1. My anxiety level in writing is higher due to the fear of making mistakes in my vocabulary/grammar.  2. I feel that my current Chinese language skills are not good enough to do the best I can with this task.  3. I feel anxious during the writing task because I do not know the right words to choose.  4. I will use the wrong words due to nervousness and anxiety. | 23 | 40 |
|  | Personal response | 1. I was a bit anxious about this task as soon as I approached it, as I had not participated in similar ones before.  2. For this task, at first, I was not sure if I would be able to complete it successfully.  3. I felt that the task was a bit complicated, and I was worried that I would not be able to complete it on time.  4. I didn’t know how to write about the writing topic at first. | 27 | 72 |
|  | Future plans | 1. I hope to improve my Chinese writing skills by taking part in this assignment.  2. I hope that I will not be nervous and anxious about taking part in future tasks. | 4 | 4 |
| Other factors | Curriculum arrangement (Chinese) | 1. I am a little dissatisfied with the current Chinese writing classes.  2. I wish Chinese writing classes were as good as this assignment.  3. I think the Chinese writing class needs to be adjusted. | 7 | 10 |
|  | COVID-19 | 1. Affected by t COVID-19, I feel that I have not had time to adequately exercise my Chinese language skills.  2. I wish COVID-19 will end soon so that I can go back to my classroom and learn Chinese with my classmates. | 3 | 3 |

*Note*: N=Number, F= Frequency
